# Researching Effective Strategies to Improve Insulin Sensitivity in Children and Teenagers - RESIST. A randomised control trial investigating the effects of two different diets on insulin sensitivity in young people with insulin resistance and/or pre-diabetes. 

**DOI:** 10.1186/1471-2458-10-575

**Published:** 2010-09-25

**Authors:** Sarah P Garnett, Louise A Baur, Manny Noakes, Katharine Steinbeck, Helen J Woodhead, Susie Burrell, Kerryn Chisholm, Carolyn R Broderick, Robert Parker, Sukanya De, Shubha Shrinivasan, Lori Hopley, Gilly Hendrie, Geoffrey R Ambler, Michael R Kohn, Chris T Cowell

**Affiliations:** 1 Kids Research Institute, The Children's Hospital at Westmead, Locked Bag 4001, Westmead NSW 2145, Australia; 2 Institute of Endocrinology and Diabetes, The Children's Hospital at Westmead, Locked Bag 4001, Westmead NSW 2145, Australia; 3 The Children's Hospital at Westmead Clinical School, University of Sydney, Locked Bag 4001, Westmead NSW 2145, Australia; 4 CSIRO Food and Nutritional Sciences, PO Box 10041, Adelaide BC SA 5000, Australia; 5 Academic Department of Adolescent Medicine, Sydney Medical School, University of Sydney NSW 2066, Australia; 6 Sydney Children's Hospital at Campbelltown, Australia; 7 Nutrition and Dietetics and Weight Management Services, The Children's Hospital at Westmead, Locked Bag 4001, Westmead NSW 2145, Australia; 8 The Children's Hospital Institute of Sports Medicine, The Children's Hospital at Westmead, Locked Bag 4001, Westmead NSW 2145, Australia; 9 Centre for Research into Adolescent's Health, The Children's Hospital at Westmead, Locked Bag 4001, Westmead NSW 2145, Australia

## Abstract

**Background:**

Concomitant with the rise in childhood obesity there has been a significant increase in the number of adolescents with clinical features of insulin resistance and prediabetes. Clinical insulin resistance and prediabetes are likely to progress to type 2 diabetes and early atherosclerosis if not targeted for early intervention. There are no efficacy trials of lifestyle intervention in this group to inform clinical practice. The primary aim of this randomised control trial (RCT) is to determine the efficacy and effectiveness of two different structured lifestyle interventions differing in diet composition on insulin sensitivity, in adolescents with clinical insulin resistance and/or prediabetes treated with metformin.

**Methods/design:**

This study protocol describes the design of an ongoing RCT. We are recruiting 108 (54 each treatment arm) 10 to 17 year olds with clinical features of insulin resistance and/or prediabetes, through physician referral, into a multi-centred RCT. All participants are prescribed metformin and participate in a diet and exercise program. The lifestyle program is the same for all participants except for diet composition. The diets are a high carbohydrate, low fat diet and a moderate carbohydrate, increased protein diet.

The program commences with an intensive 3 month dietary intervention, implemented by trained dietitians, followed by a 3 month intensive gym and home based exercise program, supervised by certified physical trainers. To measure the longer term effectiveness, after the intensive intervention trial participants are managed by either their usual physician or study physician and followed up by the study dietitians for an additional 6 months. The primary outcome measure, change in insulin sensitivity, is measured at 3, 6 and 12 months.

**Discussion:**

Clinical insulin resistance and prediabetes in the paediatric population are rapidly emerging clinical problems with serious health outcomes. With appropriate management these conditions are potentially reversible or at least their progression can be delayed. This research study is the first trial designed to provide much needed data on the effective dietary management for this cohort. This study will inform clinical practice guidelines for adolescents with clinical insulin resistance and may assist in preventing metabolic complications, type 2 diabetes and early cardiovascular disease.

**Trial registration:**

Australian and New Zealand Clinical Trials Registration Number ACTRN12608000416392

## Background

Concomitant with the rise in childhood obesity there has been a significant increase in the number of adolescents with clinical signs of insulin resistance and prediabetes [1]. It is essential that adolescents with clinical insulin resistance and prediabetes are targeted for early intervention. Unmanaged, they are likely to progress to type 2 diabetes and early atherosclerosis [2]. Development of type 2 diabetes in young people is of particular concern because complications are common and appear early in the disease [3,4].

Avoidance of progression to type 2 diabetes is of enormous benefit, not only to the individual in terms of increasing life expectancy and quality of life, but also in economic terms to society and health care payers [5]. Evidence, primarily from adult studies, indicates that progression can be reduced by diet, exercise and metformin [5].

### Diet

The role of the specific macronutrient content of the diet in the management of insulin resistance in adolescents has not been previously investigated. Most current methods of dietary management focus on weight management and recommend a high carbohydrate, low fat approach [6]. However, a recent systematic review of the dietary management of obesity in children and adolescents concluded: *"There is a marked mismatch between the public health importance of childhood obesity and the number and quality of the studies conducted so far to assess dietary interventions for weight reduction in childhood and adolescence, and little evidence to support the current recommendation of a low-fat energy-restricted diet."*[7]. The effectiveness of a low fat high carbohydrate dietary pattern over the long term remains uncertain, and despite widespread dissemination of the public health message to adopt a low fat high carbohydrate diet, rates of obesity, insulin resistance, prediabetes and type 2 diabetes continue to climb.

One alternative is a moderate carbohydrate, increased protein diet. In adults, several studies indicate that this diet may enhance weight loss and improve metabolic markers including post prandial glucose levels [8-10]. Not all studies support these findings [11], although, there are several supportive mechanisms proposed. The relative success of lower carbohydrate, higher protein diets has been related to i) an increase in satiety [12,13] ii) effects on thermogenesis, iii) favourable changes in body composition (preserves fat free mass and reduces fat mass)[14]; and iv) decreased energy-efficiency, all of which are related to protein metabolism [15-17]. However, no clinical trial data are available in children and adolescents.

Little is known about the optimal dietary intervention strategy for overweight and obese children and adolescents. Intervention studies have used hypo-caloric diets, with and without energy based food exchange system (for example Traffic Light guides), national dietary guidelines and protein sparing modified fasts, with varying results [18]. However, many inadequately described the details of the prescribed dietary intervention, making replication of the interventions difficult [18]. Structured, restrictive weight management diets have been also been described [19]. While there is general concern that this may be counter productive to the development of healthy eating habits, anecdotally, many young people who have successfully lost weight in our Weight Management Services frequently request dietary prescription. This observation supports the need for high quality studies which includes detailed dietary interventions to inform best practice.

### Exercise

Including a physical activity intervention in the management of overweight and obese adolescents is considered to be part of best practice [20]. Physical activity, with or without dietary intervention, is effective in improving body composition and may improve insulin sensitivity and other metabolic markers [21,22]. Most paediatric studies have used aerobic training; however, evidence from two small paediatric studies suggests that resistance training may also be important for improving insulin sensitivity, body composition and fat distribution [23,24].

### Metformin therapy

There are now five randomised control trials (RCTs) [25] that have demonstrated the beneficial effects of metformin in adolescents with insulin resistance and/or prediabetes. These results support previous studies assessing the role of metformin in adolescents with type 2 diabetes, polycystic ovary syndrome and non-alcoholic fatty liver disease [26-28]. The effect of metformin therapy tends to be small. Results from our systematic review and meta-analysis indicate that after 6 months of metformin therapy in children and adolescents with insulin resistance there was a statistically significant mean decrease in fasting insulin, homeostasis model assessment of insulin resistance (HOMA) and body mass index (BMI) of 9.6 microU/ml (95% CI: 6.3 to 13.0) and 2.7 kg/m^2 ^(95% CI: 1.7 to 3.6), respectively, compared to the placebo control group [25]. Results from studies in adults suggest that a lifestyle change with pharmacotherapy exceeds the benefit of either intervention alone [29]. However, no study has evaluated more intensive lifestyle interventions in conjunction with metformin in this target group.

## Study Aims

The primary aim of this study is to determine the efficacy of two different structured dietary interventions on insulin sensitivity, in adolescents with clinical evidence of insulin resistance and/or prediabetes treated with metformin. The isocaloric diets are 1) a high carbohydrate, low fat diet and 2) a moderate carbohydrate, increased protein diet.

We hypothesise that the moderate carbohydrate, increased protein diet will be more effective than the high carbohydrate diet in improving insulin sensitivity and metabolic profile in adolescents with clinical features of insulin resistance and/or prediabetes.

## Design and Methods

### Ethics approval

The study has been reviewed and approved by The Children's Hospital at Westmead (CHW) Human Research Ethics Committee (07/CHW/12), Sydney South West Area Health, Western Zone (08/LPOOL/195) and Sydney South West Area Health Service, Royal Prince Alfred Hospital (08/RPAH/455). Written informed consent from parents and assent from the young people is sought prior to their enrolment in the study.

### Design

The study is a 12 month RCT, taking place at three tertiary hospitals in Sydney, Australia: CHW (the main study centre), Royal Prince Alfred Hospital and Campbelltown Hospital. The project consists of three phases:

Phase I (0 to 3 months): Intensive structured dietary intervention

Phase II (4 to 6 months): Intensive exercise program

Phase III (7 to 12 months): Maintenance

**Figure 1:**
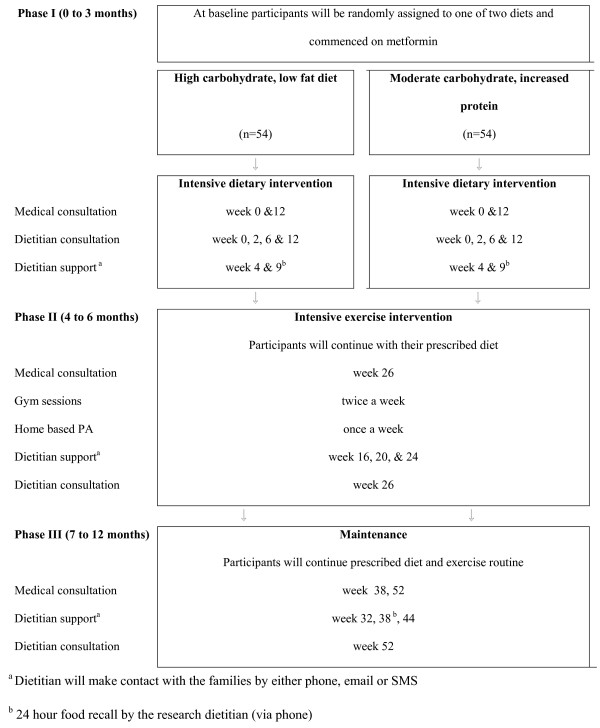


The overall schedule for the intervention is shown in Figure 1.

At the commencement of the study the children and adolescents are randomised to one of two diets. All participants are treated with metformin and receive the same overall lifestyle intervention. The only difference between the two treatment arms is the composition of the diet.

### Outcome measures

The primary outcome measure is insulin sensitivity. Insulin sensitivity is measured at baseline and after 3 and 12 months at CHW by the whole body insulin sensitivity index derived from an oral glucose tolerance test (OGTT) using the following formula: 10 000/√ ((Fasting insulin × fasting glucose) × (mean 2 hr glucose × mean 2 hr insulin))[30]. The potential confounding variables include adiposity and change in adiposity, pubertal stage, fitness and physical activity and dietary adherence. The secondary outcome measures are metabolic profile indicators including: acanthosis nigricans score, amenorrhoea/oligomenorrhoea, blood pressure, total cholesterol, HDL-cholesterol, triglycerides, waist to height ratio, hs-CRP, adipokines. Changes in outcome measures will be examined over time, as will differences between trial arms.

### Participants and recruitment

Approximately 108 (54 in each treatment arm) 10 to 17 year olds with clinical insulin resistance and/or prediabetes are being recruited through doctor referral from the three tertiary hospitals trial sites in Sydney as well as local general practitioner (primary care physician) and paediatrician referral. After a patient has been assessed and identified as meeting the trial criteria the patient and parent or carer is informed about the study by the referring doctor or study physician. If interested in participating, the patient and parent or carer makes phone contact with the research dietitian who explains the study design and sends a parent/carer information sheet and an information sheet for the adolescent detailing the study researchers, intervention, the potential benefits and required commitment and consent form. Once written consent is obtained by the study team the participant is randomised to one of two diets.

### Selection criteria

#### Inclusion criteria

Ten to 17 year olds who are overweight or obese, as defined by the International Obesity TaskForce criteria [31] with either pre type 2 diabetes as defined by the American Diabetes Association [32] and/or clinical insulin resistance are eligble for recruitment. Clinical insulin resistance is defined as overweight or obese and fasting insulin (pmol/L)/glucose (mmol/L) ratio >20 with one or more of the following acanthosis nigricans, polycystic ovarian syndrome (National Institute of Health criteria [33]), hypertension [34], fasting HDL cholesterol < 1.03 mmol/L [35] or fasting triglycerides ≥ 1.7 mmol/L [35].

#### Exclusion criteria

Type 1 or type 2 diabetes, contraindications to metformin therapy, secondary causes of obesity, psychiatric disturbance, significant mental illness, inability to take part in physical activity, weight loss medications or medications known to cause weight gain, and weight >120 kg, due to technical difficulties with dual energy x-ray absorptiometry machine (DXA). Children and adolescents taking metformin prior to commencement of the study are required to have a three month wash-out period.

### Randomisation

Treatment allocation occurs centrally by minimisation [36] with the aid of computer software [37] at CHW. To adjust for potential confounding influences participants are randomised into dietary groups stratified by sex, pubertal stage, categorised by the study physician as either prepubertal or early puberty (Tanner stage 1 and 2) or pubertal (Tanner stage 3, 4 and 5), and BMI status (overweight or obese as defined by the International Obesity TaskForce [31]).

### Sample size

The sample size is based on the primary outcome, change in whole body insulin sensitivity index (ISI) between baseline and one year, using means and standard deviations (SD) from our clinical data [38] and are consistent with published data [30]. To have an 80% chance of detecting a significant increase in whole body ISI over a one year period at the two sided 5% level, 43 young people are required in each intervention arm, assuming a clinically significant increase in whole body ISI of 0.8 and an SD of 1.3. The high SD allows for the variation of insulin sensitivity during puberty. To accommodate a 20% dropout rate we will need to recruit 54 in each intervention arm (108 in total). A 20% drop out rate is based on two Australian paediatric weight reduction trials, PEACH and HELPP, which reported a 12% and 18% [39] dropout rate at 12 months. In addition, based on our clinical experience, we anticipate a maximum of 2 or 3 participants may progress to type 2 diabetes. However, treatment for these participants is likely to be consistent with the study intervention.

### Interventions

At the commencement of the study all participants are commenced on metformin and randomised to one of two diets. The participants are encouraged to follow their prescribed diet for the duration of the trial.

#### Metformin

Metformin (Diabex) is dispensed through the pharmacy at CHW and is provided at no cost to all participants. The initial dose is 250 mg twice a day. After the first two weeks this is increased to a final dose of 500 mg twice daily.

#### Diets

Diet 1 is a high carbohydrate, low fat diet, that is 55-60% carbohydrate (moderate glycaemic load), 30% fat (≤ 10% saturated fat) and 15% protein. Diet 2 is a moderate carbohydrate, increased protein diet, that is 40-45% total energy (moderate glycaemic load), 30% fat (≤ 10% saturated fat), 25-30% protein. Both diets are prescriptive and two different energy levels are prescribed depending upon age: 6,000 to 7,000 kJ (10 to 14 year old) or 7,000 to 8,000 kJ (15 to 17 year old). Representative menus for the two diets are shown in Table 1.

**Table 1:** Representative menu for the two diets

	Diet 1 High carbohydrate, low fat	Diet 2 Moderate carbohydrate, increased protein
**Breakfast**	2 breakfast (Weet-Bix) biscuits	1 slice of wholemeal toast
	200 mL low-fat milk	1 tsp margarine
	1 tsp honey	1 egg
	1 piece fruit	1 piece fruit
		
**Morning Tea**	200 mL low-fat flavoured milk	200 g low-fat yoghurt
	30 g muesli bar	1 piece fruit
		
**Lunch**	2 slices wholemeal bread	2 slices wholemeal bread
	1 tsp margarine	1 tsp margarine
	Unlimited salad vegetables	50 g lean meat
	1 piece of fruit	Unlimited salad vegetables
		
**Afternoon tea**	2 slices wholemeal bread toasted	4 wholegrain cracker biscuits
	4 tsp peanut butter	1 slice reduced fat cheese
		
**Dinner**	1 cup cooked pasta	150 g beef steak
	100 g meat & tomato based sauce	1 potato mashed
	1 cup (or more) mixed vegetables/salad	1 cup (or more) mixed vegetables/salad
		
**Supper**	2 scoops low-fat ice cream	200 mL reduced-fat milk
	1 cup strawberries	1 tbs Milo

#### Medical care

Clinical progress is reviewed by the participant's primary referring physician or study physician at 3, 6, 9 and 12 months, unless otherwise indicated. The physician assesses pubertal staging, blood pressure, grading of acanthosis nigricans, menstrual history and metformin dosage/adherence. The physician is blinded to the trial arm of the individual and participants are requested to not inform their physician.

### Phase 1: Intensive structured dietary intervention (0 to 3 months)

Phase I assesses the impact of the macronutrient content of the diet on insulin sensitivity. The trial dietitian (lifestyle coach) delivers a standardised intervention to both trial arms, which utilises a prescribed meal plan and coaching model. Participants are not given the macronutrient content of the diets; they have been simply told that they will be randomised to one of two diets and they are given a meal plan, titled 'My Meal Plan'. The meal plans take into consideration adolescent food preferences, with the aim of weight loss. The coaching framework provides a theoretical basis for a number of key psychological variables which are important in achieving dietary compliance, lifestyle change and sustainable weight loss, such as self efficacy, stage of change intervention, autonomy, assuming responsibility, self monitoring, goal setting, accountability and self directed behaviour change[21,40].

During Phase I, food consistent with the prescribed diet plans and equating to approximately 25% of the participants energy requirements is given to the families. During this phase physical activity advice is standardised and delivered by the trial dietitian. The advice is consistent with Australian recommendations for children and adolescents including promoting an increase in incidental activity, a decrease in sedentary behaviour and an increase in active transport [41].

During this phase the participants and at least one parent/carer are expected to attend four face to face meetings with the dietitian as outlined in Figure 1. In addition to the face to face contact the dietitian also makes contact with the participant using either phone, email or text message, depending on the preference of the participant. This contact is aimed at the participant to assist with motivation and answer questions. It may or may not involve the parent/carer.

### Phase II: Intensive exercise program (4 to 6 months)

Phase II assesses the impact of a supervised, general exercise program on the treatment effect of diet and metformin. All participants receive an exercise program as well as nutrition coaching, as outlined in Figure 1. The exercise program is conducted in small groups for 1 hour, twice a week for 12 weeks in a commercial gym, Fitness First. Participants are given free access to Fitness First gyms in the geographic area in which they live. The gym program consists of a circuit training program with an age-appropriate mix of resistance exercises and aerobic stations conducted by a qualified physical trainer. In addition to the gym program participants are encouraged to exercise once a week at home. During this phase the trial dietitian offers support to the participants every 4 weeks, Figure 1.

### Phase III: Maintenance (7 to 12 months)

During Phase III participants are managed by their usual physician or study physician and followed up by the trial dietitian as indicated in Figure 1. They are encouraged to continue with their diet and exercise regimens and metformin (if required).

### Training the dietitians (lifestyle coaches)

Two dietitians are employed to specifically to work on this trial. Training of these dietitians to deliver the interventions is provided by two other experienced dietitians (SB/KC) with expertise in lifestyle counselling in children and adolescents with insulin resistance and prediabetes. Training includes shadowing in the hospital setting in order to gain experience and supervision is received on a regular basis, approximately 6 weekly intervals, as per department guidelines for training of dietitians.

### Blinding

Assessors of the main outcome measures are blinded to treatment allocation. These include the two trial clinical nurse consultants responsible for undertaking the OGTTs and anthropometric measures, the technician who takes the dual energy x-ray absorptiometry (DXA) measurements and the primary and study physicians.

### Measurements

Details of measurements are outlined below and the measurement schedule is shown in Table 2.

**Table 2:** RESIST Measurement Schedule

Measurement	Baseline	3 months	6 months	9 months	12 months
**Clinical Assessment**					
Anthropometry	✓	✓	✓		✓
Blood pressure	✓	✓	✓		✓
Pubertal staging	✓	✓	✓		✓
Acanthosis nigricans grading	✓	✓	✓		✓
**Body composition**					
Dual energy x-ray absorptiometry	✓	✓			✓
**Metabolic Measures**					
Oral glucose tolerance test	✓	✓			✓
Fasting blood sample	✓	✓	✓		✓
Urine sample (spot)	✓	✓	✓		✓
**Diet: Self monitoring, intake and questionnaires**					
Dietary checklist and questionnaires		✓	✓		✓
24 hour recall		✓	✓	✓	✓
**Physical activity, sedentary behaviour and fitness**					
CLASS questionnaire	✓	✓	✓		✓
Fitness testing	✓		✓		✓
**Metformin adherence**					
Pill count		✓	✓	✓	✓

#### Clinical Assessment

##### Family background and family medical history

Demographic and family medical history information is collected at baseline by the study physician.

##### Anthropometry

Weight and height are measured using standard procedures [42]. For weight a single measurement, measured to the nearest 0.1 kg, is recorded and used for data analysis. Height is measured twice, to the nearest 0.1 cm, and the average value is used for data analysis. Waist circumference is measured to the nearest 0.1 cm using a flexible steel tape. The waist is defined as the horizontal distance around the umbilicus using the left hand under technique. The average of three measurements will be used for data analysis. Waist to height ratio will be used as a measure of central adiposity [43].

##### Pubertal status

Pubertal status is categorised according to the Tanner Scale after assessment by the study physician [44].

##### Blood Pressure

Blood pressure is measured using an automated blood pressure monitor (Dinamap 1846 SX) according to standard procedures [45].

##### Acanthosis nigricans

Acanthosis nigricans in the axilla and neck is graded by the study physicians using the Burke scale [46].

#### Body composition

##### Dual energy x-ray absorptiometry (DXA)

DXA (Prodigy, Lunar-GE, Madison, WI USA) equipped with propriety software version 8.6 is used to measure body composition. The manufacturer recommended scan mode (as determined by height and weight) is used for total body mass measurements. When possible, standard positioning techniques is used. Where the participant width exceeds maximum scan width, subjects are "mummy wrapped", with arms placed in a lateral position to reduce subject width. Scans are analysed using manufacturer recommended techniques. In subjects where delineation of body subregions is compromised due to overlapping of tissue from two regions, lines are positioned midway through the intersection of the two regions.

#### Metabolic measures

##### OGTT

An OGTT is performed after an overnight fast. The dose of glucose is 1.75 g/kg of body weight to a maximum of 75 g. Plasma glucose and insulin is sampled every 30 minutes for 2 hours as previously described [38].

##### Blood samples

In addition to glucose and insulin, blood drawn at time 0 (OGTT) is analysed using standard techniques for full blood count, lipids (total cholesterol, HDL-C, triglycerides,), hs-CRP, oestradiol, testosterone, sex hormone binding globulin, lactate, hepatic transaminases (alanine aminotransferase, gamma-glutamyl transferase), renal function tests (urea, electrolytes, and creatinine). Blood (plasma and serum) is stored at -80°C in the Institute of Endocrinology and Diabetes, CHW for future analysis of homocystine (plasma), adipokines and vitamin B12.

##### Urine samples

Spot urine samples are collected and analysed for albumin to creatinine ratio using standard techniques.

#### Diet: Self monitoring, intake and eating behaviours

##### Diet checklist

Seven day diet checklists are completed by the participants for self monitoring, with assistance from the parent or guardian if required. The checklists were adapted from those used in previous studies [9].

##### Dietary 24 hour recall

Dietary intake is assessed using a methodology adapted from USDA five pass method as used in the 2007 Australian National Children's Nutrient and Physical Activity Survey [47]. In brief, at each time point (weeks 9, 12, 26, 38 and 52) food and beverage intake is measured using a three pass 24 hour dietary recall. To assist with estimation of portion sizes a food model book is provided with picture guides of common household measures and life size images, plates, bowls, glasses and amorphous dishes.

##### Eating behaviour

Eating behaviour is assessed by the Eating Pattern Inventory for Children [48].

##### Qualitative aspects of the diet

Qualitative aspects of the diet including, perceptions of cost, satiety, ease of compliance are assessed by a locally developed questionnaire. This questionnaire is completed by the parent or guardian.

#### Physical activity, sedentary behaviour and fitness

##### Physical activity and sedentary behaviour

The Children's Leisure Activities Study Survey (CLASS) is completed by the participant with assistance from the parent or guardian if required. CLASS assesses a range of physical activity and sedentary behaviours. CLASS has been validated for use among children, and used by adolescents aged 13-16 years in the Nepean Study [49]

##### Fitness

Assessment of aerobic fitness is performed using a Bruce treadmill protocol and breath by breath gas analysis. VO_2 _peak (maximal oxygen uptake) is expressed relative to body mass [50].

#### Metformin compliance

Metformin adherence is assessed by pill counts by the clinical trials pharmacist at CHW.

### Data analysis

Data analyses will be carried out according to a pre-established plan, with the primary analysis based on intention to treat and with statistical support from the Clinical Epidemiology Unit, CHW. Analysis will be undertaken blinded, at the end of each phase of intervention. Generalised linear models using a repeated measures option will be used to assess differences in continuous variables between baseline and follow up by treatment group after adjusting for baseline covariates if necessary [51]. Mixed models will also be used to investigate changes over time. The mixed models procedure will take into account within subject correlations and missing data points. A time by group interaction will be included to investigate whether rates of change of adiposity and other primary outcomes are significantly different between the two study groups. In addition, the proportions of young people who were successful in decreasing adiposity will be compared between groups and the result expressed as number-needed-to-treat.

## Discussion

Clinical insulin resistance and prediabetes in the paediatric population are rapidly emerging clinical problems with serious health outcomes. With appropriate management these conditions are potentially reversible or at least their progression can be delayed. This research study is the first trial designed to provide definitive data on the effective dietary management for this cohort. This is crucial information to prevent metabolic complications, type 2 diabetes and early cardiovascular disease.

## Funding

Diabetes Australia Research Trust 2008, BUPA Health Foundation Australia Pty Limited (formerly MBF Foundation) 2008 to 2011, Heart Foundation (#G 08 S 3758), 2009 to 2010. Sarah Garnett is supported by a National Health and Medical Research Council Australian Clinical Research Fellowship (#457225).

## Competing interests

The authors declare that they have no competing interests.

## Authors' contributions

SPG, CTC, LAB participated in all aspects of the conception, design and funding of the study. MN participated in all aspects of the design of the study, with particular emphasis on design of the dietary intervention, and funding. KS, HJW, SS, GRA, MRK, SD provided medical expertise and design of the study. RP and CB were responsible for the development of the exercise intervention and testing. SB, KC and LH were responsible for the development of the dietary intervention. GH was responsible for the methodology of the 24 hour recall. All authors were responsible for drafting the manuscript and have read and approved the final manuscript.

## Pre-publication history

The pre-publication history for this paper can be accessed here:

http://www.biomedcentral.com/1471-2458/10/575/prepub
